# Isolation of Polysaccharides from *Trichoderma harzianum* with Antioxidant, Anticancer, and Enzyme Inhibition Properties

**DOI:** 10.3390/antiox10091372

**Published:** 2021-08-28

**Authors:** Kandasamy Saravanakumar, SeonJu Park, Anbazhagan Sathiyaseelan, Arokia Vijaya Anand Mariadoss, Soyoung Park, Seong-Jung Kim, Myeong-Hyeon Wang

**Affiliations:** 1 Department of Bio Health Convergence, Kangwon National University, Chuncheon 200-701, Korea; saravana732@kangwon.ac.kr (K.S.); sathiyaseelan@kangwon.ac.kr (A.S.); drmavanand@kangwon.ac.kr (A.V.A.M.); 202116272@kangwon.ac.kr (S.P.); 2 Chuncheon Center, Korea Basic Science Institute (KBSI), Chuncheon 24341, Korea; sjp19@kbsi.re.kr; 3 Department of Physical Therapy, College of Health and Science, Kangwon National University, Samcheok-si 24949, Korea

**Keywords:** antioxidant, antidiabetics, polysaccharides, MDA-MB293, cytotoxicity, *Trichoderma*

## Abstract

In this work, a total of six polysaccharides were isolated from culture filtrate (EPS1, EPS2) and mycelia (IPS1–IPS4) of *Trichoderma harzianum*. The HPLC analysis results showed that EPS1, EPS2, IPS1, and IPS2 were composed of mannose, ribose, glucose, galactose, and arabinose. The FT-IR, ^1^H, and ^13^C NMR chemical shifts confirmed that the signals in EPS1 mainly consist of (1→4)-linked α-d-glucopyranose. EPS1 and IPS1 showed a smooth and clean surface, while EPS2, IPS2, and IPS3 exhibited a microporous structure. Among polysaccharides, EPS1 displayed higher ABTS^+^ (47.09 ± 2.25% and DPPH (26.44 ± 0.12%) scavenging activities, as well as higher α-amylase (69.30 ± 1.28%) and α-glucosidase (68.22 ± 0.64%) inhibition activity than the other polysaccharides. EPS1 exhibited high cytotoxicity to MDA-MB293 cells, with an IC_50_ of 0.437 mg/mL, and this was also confirmed by cell staining and FACS assays. These results report the physicochemical and bioactive properties of polysaccharides from *T. harzianum*.

## 1. Introduction

Glycobiology is a division of science that elucidates the biosynthesis and structural characteristics of saccharides and glycoconjugates [1]. The connective bonding of monosaccharide subunits forms the increasingly complex structure of polysaccharides through glycosidic linkages [2]. In general, the polymers are classified as glycoconjugates (proteoglycans, glycolipids, and glycoproteins), homopolysaccharides (cellulose, starch, inulin, chitin, pectins, and glycogen), and heteropolysaccharides (hyaluronic acid, chondroitin-4-sulfate, gamma globulin, and heparin) [1]. Plants, microbes (bacteria, fungi, and yeast), animals, algae, and lichens are considered promising natural resources for the isolation of bioactive polysaccharides [3]. These polysaccharides have gained astonishing attention in biomedicine due to their unique biochemical properties, as well as their antioxidant and immunomodulatory effects [4,5].

The fungal cell wall is 90% composed of polysaccharides, including glycogen, β-glucan, α-glucan, galactan, mannan, galactomannan, xylomannan, polygalactosaminde, chitin, chitosan, cellulose, and polyuronide [6]. These polysaccharides play a predominant role in the rheological, growth, pathogenicity, cell protection, resistance, chemical signal transfer, and stress-related pathways of fungi [7]. Recently, fungal polysaccharides have gained great attention for pharmacological, cosmetic, and food applications due to their biological properties (e.g., antiviral, antimicrobial, antitumor, antioxidant, immunomodulatory, hypolipidemic, hepatoprotective, and hypoglycemic), biodegradability, and biocompatibility [8,9,10,11]. Furthermore, fungal polysaccharides are used in nanoscience and tissue engineering as nanoparticles, drug carriers, and bone regeneration materials [1,12]. For instance, fungal chitosan is used as a base material for the preparation of wound dressing materials [13]. 

The ubiquitous fungi *Trichoderma* (Ascomycota, *Hypocreales*, *hypocreaceae*) contain ~250 species. The fungal genus *Trichoderma* is plentifully isolated from water, soil, roots, decaying wood, and healthy plant tissue [14,15,16]. *Trichoderma* species are well reported as biocontrol agents used as biofertilizers in agricultural crop cultivation to inhibit plant pathogens and trigger plant immunity [17,18]. This fungus is also known to produce various molecules, including enzymes and secondary metabolites with promising anticancer, antitumor, antioxidant, antibacterial, and antiviral activities [19,20,21,22,23,24]. However, only a few works have reported on the polysaccharides from *Trichoderma* species with anticancer, microphage activation, and antioxidant activities [3,25,26,27,28,29]. In addition, the burgeoning of novel chronic disorders, including cancer and oxidative-stress-related diseases, prompts the search for novel health-beneficial compounds from plants, fungi, and bacteria. Therefore, this work aimed to isolate and purify intracellular polysaccharides (IPSs) and extracellular polysaccharides (EPSs) from *T. harzianum* and evaluate their antioxidant, enzyme inhibition, and anticancer activity. 

## 2. Materials and Methods

### 2.1. Extraction and Characterization of Polysaccharides 

#### 2.1.1. Isolation of Crude IPSs and EPSs

The materials used in the present study are summarized and shown in the Supplementary Materials. The IPSs and EPSs were isolated from *T. harzianum* according to the methods described previously, with minor modifications [25,29]. For the preparation of seed culture, *T. harzianum* was inoculated in potato dextrose agar and incubated at 28 ± 2 °C for 4 days. The actively growing edge of *T. harzianum* (7 mm plugs) was aseptically inoculated in 2 L of potato dextrose broth in a 5 L Erlenmeyer flask and incubated in a shaking incubator at 28 ± 2 °C and 180 rpm for 10 days. Afterwards, the culture supernatant and mycelium were collected by centrifugation at 10,000 rpm for 10 min. The mycelium and supernatant were collected and used for the isolation of IPSs and EPSs, respectively. The IPSs were ultrasonically extracted from the mycelia using distilled H_2_O (1:10 w/v) for 10 min, and then boiled at 120 °C for 4 h. Afterwards, the extract was concentrated by using a rotary evaporator at 40 °C and precipitated with a triple volume of 95% ethyl alcohol at 4 °C overnight. The precipitates were collected by centrifugation at 8000 rpm for 10 min. The collected precipitate of IPSs was decolorized and deproteinized using the Sevage solution composed of chloroform: *n*-butyl alcohol (4:1) [30]. After this treatment, the aqueous phase containing the IPSs was collected, dialyzed, freeze-dried, labeled as crude IPSs, and stored at 4 °C. Moreover, for the EPS isolation, the culture supernatant was freeze-dried and the dried culture supernatant was mixed with a triple volume of 95% ethyl alcohol (w/v) and incubated at 4 °C overnight. The precipitate was then collected, deproteinized with Sevage solution treatment, dialyzed, freeze-dried, labeled as crude EPSs, and stored at 4 °C. 

#### 2.1.2. Separation and Purification of IPSs and EPSs

The IPSs and EPSs were purified using the DEAE Sepharose Fast Flow column (1.6 × 20 cm) according to the methods reported previously [25]. First, 1 g of IPSs or EPSs was dissolved in 20 mL of distilled H_2_O, mixed well, centrifuged at 8000 rpm for 10 min, and filtered using 0.45 µm filter paper. Then, 5 mL of the filtrate was loaded in the DEAE Sepharose Fast Flow column with 0–0.5 M NaCl as a gradient to elute the polysaccharides at a flow rate of 3 mL/10 min. The fractions were collected in response to NaCl concentration and detected by phenol–sulfuric acid assay [31]. Finally, the collected fractions were pooled together based on the gradient solution. Furthermore, the purity of the polysaccharides (IPSs and EPSs) was determined by subcolumn using the Sephacryl S-300 HR column (1.6 × 60 cm) with a flow rate of 1 mL/min. The purified fractions of six polysaccharides were named EPS1, EPS2, IPS1, IPS2, IPS3, and IPS4. 

#### 2.1.3. Chemical Composition Analysis

The total carbohydrates were measured by phenol–sulfuric acid assay [31]. The protein content in polysaccharides was determined using Coomassie Brilliant Blue G-250 against a standard solution of bovine serum albumin [32]. Total phenol content (TPC) was measured using the Folin–Ciocalteu method. and total flavonoid content (TFC) was measured via colorimetric methods [33]. The nucleic acids and proteins were also detected using the NanoDrop system by scanning at the range of 190–540 nm [25]. 

#### 2.1.4. Monosaccharide Composition

The monosaccharide composition was determined according to the detailed protocol described previously [34], with slight modifications. First, 10 mg of each polysaccharide (EPS1, EPS2, and IPS1-4) was dissolved in 8 mL of 2 M trifluoroacetic acid (TFA) and hydrolyzed for 6 h at 110 °C. Then, 200 µL of hydrolyzed polysaccharides or monosaccharide standard mix was added to 240 µL of NaOH (0.3 M). Then, a 240 µL methanolic solution of 1-phenyl-3-methyl-5-pyrazolone (PMP, 0.5 M) was added and thoroughly mixed using the vortex mixer for 10 s, then incubated at 70 °C in the oven for 2 h. After the incubation, the mixture was neutralized by adding 240 µL of HCl (0.3 M) and cooled to 25 °C. The mixture was subjected to chloroform extraction three times to remove the impurities. The chloroform layer was discarded, and the aqueous layer was collected and filtered through 0.22 µm filter paper. The filtrate was subjected to analysis using HPLC equipped with a UV detector. The mobile phase composed of 0.05 M phosphate buffer and acetonitrile (84:16, v/v) with a flow rate of 1 mL/min, column temperature of 40 °C, and injection volume of 20 µL, along with the monosaccharide standard containing sugars (d-(+)-Galactose, d-(+)-Glucose, d-(+)-Mannose, d-(+)-Xylose, d-(−)-Ribose, and d-(−)-Arabinose), were used. 

#### 2.1.5. FT-IR and NMR Analysis

The presence of the functional group in the polysaccharides (EPS1, EPS2, and IPS1-4) was determined by Fourier transform infrared (FT-IR) spectroscopy (PerkinElmer Paragon 500, Waltham, MA, USA) analysis using the KBr pellets of polysaccharides. Then, the linkage of the polysaccharides was determined by ^1^H, ^13^C and 2D NMR analysis. A total of 20 mg of each polysaccharide (EPS1, EPS2, and IPS1-4) was dissolved in 0.5 mL of D_2_O at ambient temperature. The NMR was recorded using an FT-NMR spectrometer (Bruker, 600 MHz, Fällanden, Switzerland).

### 2.2. Antioxidant Assay

#### 2.2.1. DPPH Scavenging Activity

The free radical DPPH scavenging activity of the polysaccharides (EPS1, EPS2, and IPS1-4) was determined according to the protocol described in a previous work [35]. Firstly, the DPPH (100 µL) was prepared in 99.8% methyl alcohol. Secondly, different concentrations of each polysaccharide were prepared using distilled H_2_O. Thirdly, the reaction was allowed by mixing a 1:1 ratio of polysaccharides and DPPH at ambient temperature in dark conditions for 10 min to determine the scavenging ability of polysaccharides. After completion of the reaction period, the reaction mixture was measured at 517 nm using a UV spectrophotometer. The OD values were substituted with the formula described previously to determine the percentage of DPPH scavenging [36].

#### 2.2.2. ABTS^+^ Scavenging Activity

The free radicals of the ABTS^+^ scavenging ability of the polysaccharides (EPS1, EPS2, and IPS1-4) were analyzed by following the protocols described previously [37]. Firstly, the cationic form of ABTS^+^ was prepared by mixing a 1:0.5 ratio of ABTS (7 mM) and potassium persulfate (2.45 mM) and kept in a dark room environment for 24 h. The optical density of ABTS^+^ was adjusted to 0.7 ± 0.02 at 734 nm in the UV spectrophotometric analysis by using 50% ethyl alcohol. Secondly, the scavenging reaction was activated by mixing a 1:1 ratio of ABTS^+^ and polysaccharide samples in a dark room for 10 min. Finally, the optical density of the reaction mixture was observed at 734 nm using a UV spectrophotometer, and the ABTS^+^ scavenging (%) was calculated using the formula reported previously [38].

### 2.3. Enzyme Inhibition Assay

#### 2.3.1. α-Amylase Inhibition Activity

The α-amylase inhibition ability of the polysaccharides (EPS1, EPS2, and IPS1-4) was tested according to the methods reported elsewhere [39]. In brief, different concentrations of each polysaccharide were prepared using distilled H_2_O. Subsequently, 2 U/mL of α-amylase was prepared in sodium phosphate buffer (pH 7.0) while 0.5% starch and 0.1 M NaCl were prepared in deionized H_2_O. The DNS was prepared according to the composition reported in our earlier work [38]. For the enzyme inhibition assay, 50 µL of any one of the polysaccharides was mixed with 150 µL of starch (0.5%) in 96-well plates containing 10 µL of α-amylase (2 U/mL) and incubated at 37 °C for 30 min. Later, the reaction was terminated by the addition of 20 µL of NaOH (2 M), and then 20 µL of DNS was added and the mixture was allowed to cool at room temperature. Finally, the optical density was measured at 540 nm using a UV spectrophotometer, and the α-amylase inhibition (%) was calculated using the formula reported previously [36]. 

#### 2.3.2. α-Glucosidase Inhibition Activity

The α-glucosidase inhibition of the polysaccharides (EPS1, EPS2, and IPS1-4) was analyzed following the method described elsewhere [40]. The α-glucosidase inhibition was tested by mixing a 1:1 ratio of any one of the polysaccharides and ρ-nitrophenyl glucopyranoside (5 M) and incubated at 37 °C for 30 min. Later, the reaction was terminated by the addition of 100 µL of Na_2_CO_3_ (0.1 M). Then, the absorbance of the reaction mixture was measured at 405 nm using a UV spectrophotometer, and the α-glucosidase inhibition (%) was calculated using the formula described previously [36].

### 2.4. Antiproliferation Assay

#### Cytotoxicity

The antiproliferative effects of the polysaccharides (EPS1, EPS2, and IPS1-4) were tested in cancerous and noncancerous cell lines using the WST-1 kit. For the assay, the cell suspensions (1 × 10^6^) of NIH3T3 and MDA-MB293 were prepared in DMEM (Thermo Fisher Scientific, Waltham, MA, USA) and RPMI (Thermo Fisher Scientific, Waltham, MA, USA) media, respectively, and these media were previously incorporated with 10% FBS and 1% antibiotics (penicillin–streptomycin). The 100 µL/well cell suspension of these cells was seeded in the 96-well plates and incubated in a CO_2_ environment at 37 °C for 24 h. After the incubation, 10 µL of different concentrations of any one of the polysaccharides was added, and again incubated for 16 h. Later, 10 µL of WST-1 was added to each well and again incubated in a CO_2_ environment at 37 °C for 30 min, and then the optical density was measured at 450 nm using a UV spectrophotometer. The cell viability or cytotoxicity (%) was calculated by applying the formula described elsewhere [21]. The antiproliferative effect of the polysaccharides was further confirmed by fluorescent staining assay and flow cytometry (apoptosis assay kit: Annexin V FITC; and PI kit: Thermo Fisher Scientific, Waltham, MA, USA) as reported in our previous work [38].

## 3. Results and Discussion 

### 3.1. Isolation and Physicochemical Characterization of Polysaccharides

#### 3.1.1. Extraction, Fractionation, and Characterization 

**Figure 1:**
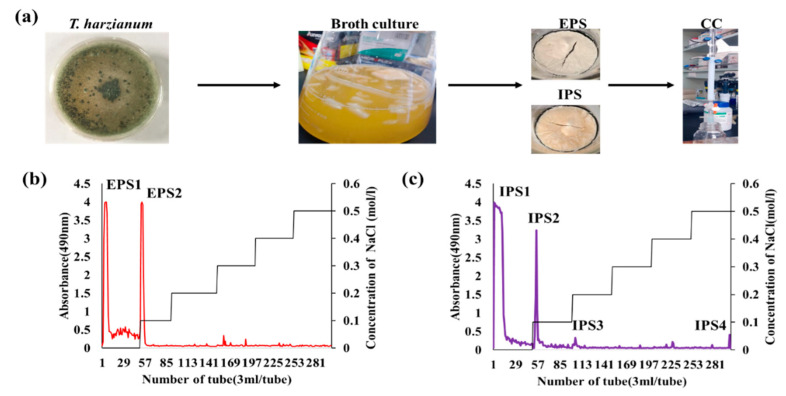
Extraction and purification of the intracellular (IPS) and extracellular (EPS) polysaccharides from the mycelium and culture filtrate of *T. harzianum* by column chromatography (CC) (**a**), DEAE Sepharose fast flow chromatography-based fractionation of EPS (**b**), and IPS (**c**) using NaCl as a gradient mobile phase.

The IPSs and EPSs were isolated from the cell culture filtrate and mycelia of *T. harzianum*, respectively. The isolated crude polysaccharides were decolorized and deproteinized, then fractionated using the DEAE Sepharose fast flow column, followed by the Sephacryl S-300 HR column using NaCl (0–0.5 M) as a gradient elute (Figure 1a). The fractions were collected and pooled based on NaCl concentration and phenol–sulfuric acid assay [31]. A total of two fractions for EPSs and four fractions for IPSs were obtained (Figure 1b,c). The weight, yield, and protein content significantly varied between the polysaccharide samples, but total phenol, total flavonoids, and nucleic acids were not found in the samples (Table 1). The highest weight was observed for EPS2, with a yield of 16.32%, while the lowest weight was found for IPS4, with a yield of 0.57% (Table 1). The protein content ranged from 0.18 ± 0.05% to 0.81 ± 0.07%, and it was found to be highest in IPS3 and lowest in IPS4 (Table 1). These results are consistent with those of an earlier work that reports the presence of protein in the polysaccharides isolated from fungi [41]. 

#### 3.1.2. Monosaccharide Composition 

**Figure 2:**
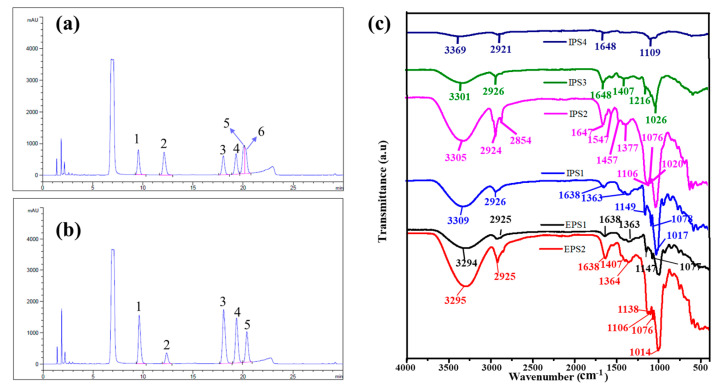
Determination of monosaccharides by HPLC using the UV detector: standard chromatogram of monosaccharide mix contains mannose (1), ribose (2), glucose (3), galactose (4), xylose (5), and arabinose (6) (**a**); determination of monosaccharides in EPS1 (**b**); and FTIR spectral analysis of EPSs and IPSs (**c**). The raw data of FTIR analysis are provided in the Supplementary Materials (SI. FTIR of EPSs and IPSs as zip file).

The monosaccharide composition of the polysaccharides was studied by the cleavage of glycosidic linkages via acid hydrolysis, followed by HPLC analysis [34]. The HPLC-equipped UV detector with a mobile phase of acetonitrile and phosphate buffer is a promising method for measuring the sugars [42]. Therefore, the monosaccharide composition of the polysaccharides (IPSs and EPSs) was determined using HPLC–UV by matching the retention time of the standard sugars with the peaks observed in the polysaccharide samples, and the results are shown in Figure 2a,b and Figure S1). The samples EPS1, EPS2, IPS1, and IPS2 displayed retention peaks corresponding to mannose, ribose, glucose, galactose, and arabinose. Meanwhile, IPS3 and IPS4 showed the retention peaks corresponding to mannose, glucose, and galactose, but the peaks for arabinose and ribose were absent Figure 2a,b and Figure S1)). These monosaccharide contents significantly varied between the polysaccharide samples (*p* < 0.05). The mannose content was found to be highest in IPS3 (35.69%) and lowest in IPS2 (20.04%). Compared to the other monosaccharides, the ribose content was found to be lower than that of other sugars. The glucose levels were found to be highest in EPS2 (40.62%) and lowest in IPS4 (21.22%). The levels of galactose were found to be highest in IPS4 (34.79%) and lowest in IPS1 (11.51%). These results indicate that the levels of these monosaccharides were better distributed in EPS1 than in the other polysaccharide fractions. Similarly, several previous works have reported the presence of intracellular monosaccharides such as glucose and mannose isolated from *Trichoderma* sp. [25], but the present work is the first to observe the presence of arabinose and ribose in both intracellular and extracellular polysaccharides derived from *T. harzianum*.

#### 3.1.3. FT-IR Analysis

The chemical functional group characteristics of the purified polysaccharides (EPS1, EPS2, and IPS1–4) were determined by FTIR analysis (Figure 2c). All six polysaccharides exhibited a strong vibration peak at 3200–3400 cm^−1^, accounting for the O–H stretching vibration of the hydrogen bond. In addition, these polysaccharides displayed C–H vibration at 2920–2940 cm^−1^ due to the establishment of acyclic saturated hydrocarbons [43]. A peak was observed at 1363–1457 cm^−1^ in EPS1, EPS2, IPS1, IPS2, and IPS3 corresponding to the methylene/alcohol group (C–H, O–H bending) [34] but IPS4 did not show the same peak. Moreover, the peaks at 1000–1300 cm^−1^ are reportedly the characteristic peaks of C–O stretching for primary or secondary alcohols in the polysaccharides [34]. Similarly, the present study also found the characteristic carbohydrate peak in all six of the polysaccharide samples (Figure 2c), confirming the polysaccharide nature of the isolated EPSs and IPSs from *T. harzianum*. Furthermore, the peaks at 847 cm^−1^, 844 cm^−1^, and 848 cm^−1^ in EPS1, EPS2, and IPS1, respectively, indicated the sugar linkage attributable to mannose residue [44,45]. These results confirm the polysaccharide nature of the EPSs (EPS1 and EPS2) and IPSs (IPS1–4) isolated from *T. harzianum*.

#### 3.1.4. NMR Analysis

**Figure 3:**
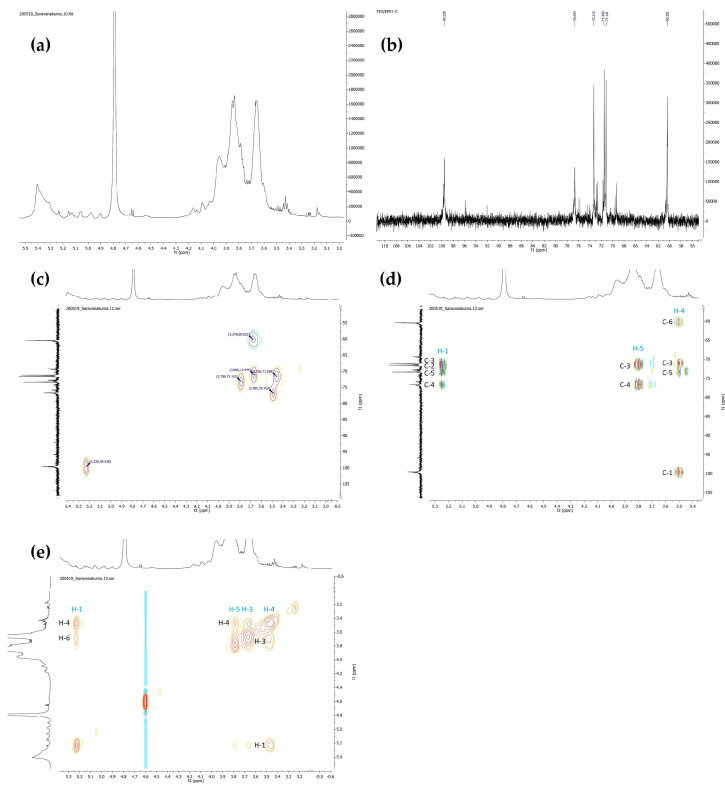
Structural characterization of EPS1 by NMR spectral analysis: ^1^H NMR spectrum (**a**), 13C NMR spectrum (**b**), HSQC spectrum (**c**), HMBC spectrum (**d**), and NOESY NMR spectrum (**e**). For better readability, refer to the Supplementary Materials (Supplementary Figure S4).

All six polysaccharides were subjected to the NMR analysis but, except for EPS1, did not show sufficiently strong signals to correlate the polysaccharide characteristics based on NMR data (Figures S2 and S3). EPS1 showed the most promising bioactivity, and a strong peak response for ^1^H, ^13^C, HMBC, HSQC, and NOESY (Figure 3). Unfortunately, although the HPLC and FTIR results showed the presence of four sugars in EPS1, the NMR signal displayed one strong sugar moiety with several weak sugars. Due to its high bioactive potential, the present work exclusively characterized EPS1 based on NMR data. The glucopyranoside assignment of signals in the ^1^H and ^13^C NMR spectra of EPS1 was achieved using two-dimensional homonuclear COSY and heteronuclear HSQC and HMBC analysis (Figure 3). The signals of EPS1 in the ^1^H NMR and ^13^C NMR chromatograms were compared with data from the literature [46,47]. The ^1^H NMR spectrum exhibited proton resonance at δH 3.3–5.5 ppm, and deduced the α-linkage of the coupling constant (*J* = 3.9 Hz) of the anomeric proton signal at δH 5.23 ppm. 

The ^13^C NMR spectrum demonstrated more chemical information than that of ^1^H NMR, with a total of six major signals at δc 99.6, 76.8, 73.1, 71.6, 71.1, and 60.0 attributed to C-1–C-6 of the glucopyranose units for EPS1. The downward chemical shifts of C-4 (δc 76.8) indicated another sugar moiety attached at C-4. The intense signal of anomeric carbon at δc 99.6 was similar to that at δc 76.8. It was proposed that the backbone was composed of →4)-α-d-Glc-(1→ units. The other four signals were assigned to the non-substituted C-2, C-3, C-5, and C-6 of the sugar moiety. The HMBC spectrum further indicated the correlations between proton and carbon signals with the →4)-α-d-Glc-(1→-linked α-d-glucopyranose residues. The ^1^H and ^13^C NMR chemical shifts are summarized in Table 2 in comparison with previous work [25]. Because of the strong signals of (1→4)-linked α-d-glucopyranose, other correlation signals were relatively weak, and hard to assign. 

#### 3.1.5. Morphology of Polysaccharides 

**Figure 4:**
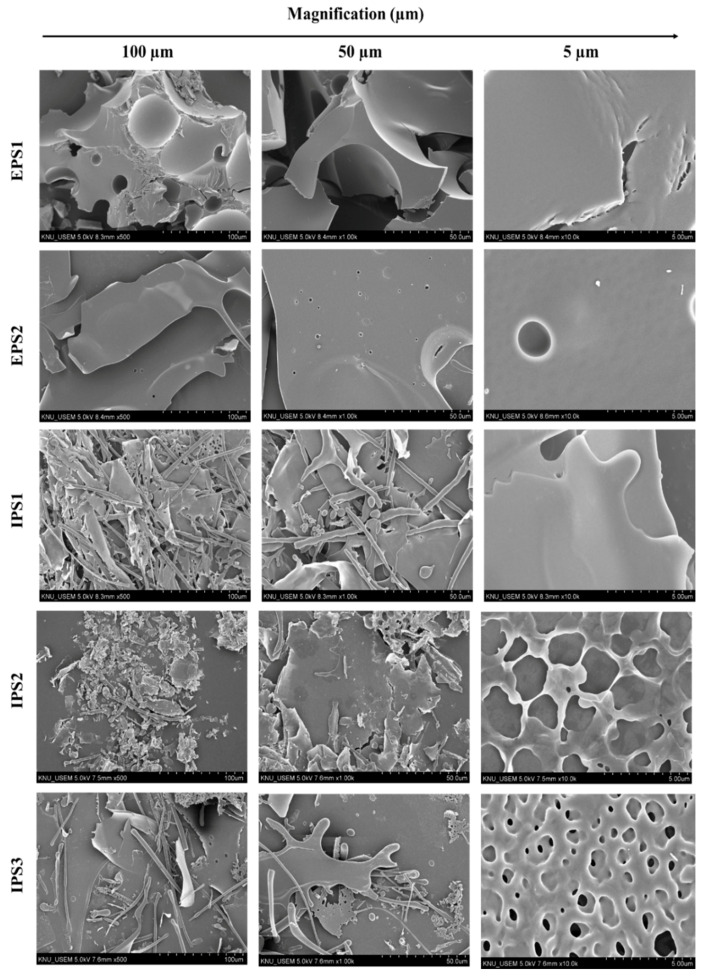
Scanning electron microscopic observation of the surface morphology of EPSs (1 and 2) and IPSs (1–3) at different magnifications (100 µm, 50 µm, and 5 µm).

The surface morphology of the polysaccharides (EPS1, EPS2, and IPS1–3) isolated from *T. harzianum* was observed under the scanning electron microscope (SEM, Figure 4). The results presented the surface morphology of polysaccharides at three different magnifications (100 µm, 50 µm, and 5 µm). All of the polysaccharides were observed to be of irregular, large, lamellar shapes, but EPS1 and IPS1 showed a smooth and clean surface, whereas EPS2, IPS2, and IPS3 exhibited a microporous polymer structure when observed at a magnification of 5 µm (Figure 4). These results also indicate the amorphous nature of EPS2, IPS2, and IPS3. Thus, EPS1 isolated from *T. harzianum* was unique in terms of its structural and bioactive characteristics, and deserves further analysis. The smooth surface of IPS1–IPS3 was found to significantly vary between the magnifications. For example, IPS1–IPS3 showed a rough and uneven surface at 100 µm magnification, but a smooth surface at 5 µm; this indicates that the nature of the polysaccharides’ surface was significantly affected by the freeze-drying process. 

### 3.2. Biological Activity 

#### 3.2.1. Antioxidant Activity 

**Figure 5:**
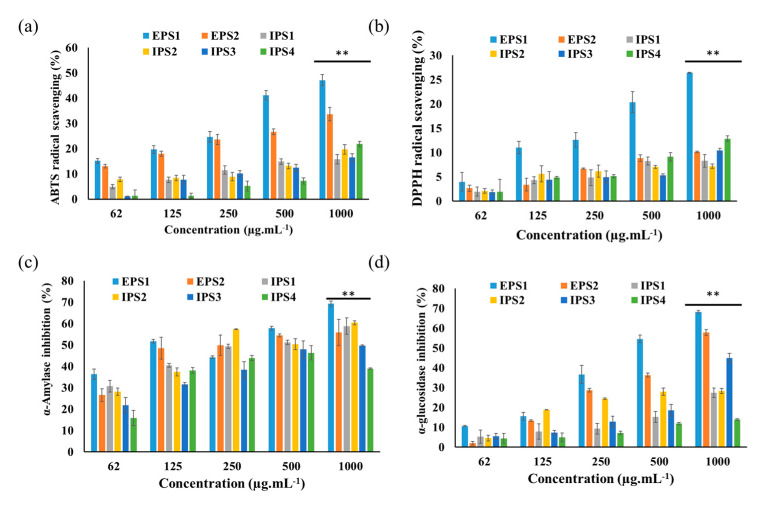
Antioxidant and enzyme inhibition activity of polysaccharides (EPSs and IPSs) isolated from *Trichoderma* spp: ABTS+ scavenging activity (**a**), DPPH free radical scavenging activity (**b**), α-amylase inhibition activity (**c**), and α-glucosidase inhibition activity (**d**). Results presented as mean ± SE (DF-2), ** *p* < 0.01 indicates the significance between the polysaccharides and their low concentration.

The natural molecules scavenge free radicals by adopting either single-electron transfer, hydrogen atom transfer approach, or both mechanisms. Moreover, the antioxidant activity of the natural molecules varies in response to the adaptive mechanism [48]. It is essential to test the natural molecules via multiple assays; hence, the present study attempted to determine the antioxidant activity of polysaccharides (EPS1, EPS2, and IPS1–4) by testing them against two free radicals—DPPH and ABTS+—by spectrophotometric assay. It was observed that all of the polysaccharides exhibited significant ABTS+ and DPPH scavenging activities in a concentration-dependent manner (Figure 5a,b). Among the tested polysaccharides, the higher concentration (1 mg/mL) of EPS1 displayed higher ABTS+ (47.09 ± 2.25%) and DPPH (26.44 ± 0.12%) scavenging activity than that of the other polysaccharides (EPS2 andf IPS1–IPS4). Similarly, the polysaccharides derived from plants (*Sophorae tonkinensis* Radix) and edible mushrooms (*Sparassis latifolia* and *Coprinus comatus*) are known to scavenge the free radicals [48,49]. Moreover, the polysaccharides from *Trichoderma* species are reported to scavenge H_2_O_2_ in a dose-dependent manner [25]. 

#### 3.2.2. Enzyme Inhibition Activity 

Enzymes such as α-amylase and α-glucosidase are involved in carbohydrate metabolism, and their inhibition regulates metabolic changes, prevents higher blood sugar elevation, and reduces the risk of diabetes [50,51]. Therefore, the present work tested the enzyme inhibition activity of the polysaccharides (EPS1, EPS2, and IPS1–4) isolated from *T. harzianum* via spectrophotometric assay. All of the polysaccharides showed significant enzyme inhibition activity with the increase in dose (*p* < 0.01). Among the polysaccharides, EPS1 (1 mg/mL) showed higher α-amylase (69.30 ± 1.28%) and α-glucosidase (68.22 ± 0.64) inhibition activity than the other polysaccharides (Figure 5c,d).

#### 3.2.3. Antiproliferative Activity 

**Figure 6:**
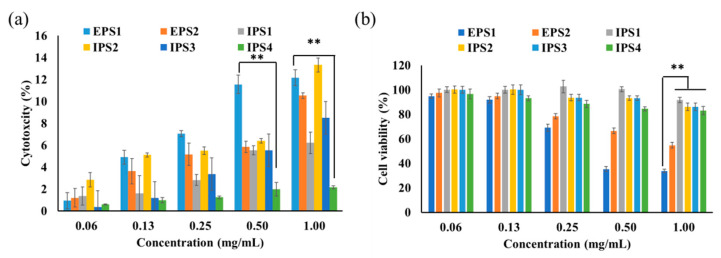
Cytotoxicity of polysaccharides (EPSs and IPSs) isolated from *Trichoderma* spp. in a noncancerous murine fibroblast (NIH3T3) cell line (**a**) and a human triple-negative breast cancer (MDA-MB231) cell line (**b**). Results presented as mean ± SE (DF-2), ** *p* < 0.01 indicates the significance between the polysaccharides and their low concentration.

The polysaccharides of *T. harzianum* did not show significant cytotoxicity (>15%) to the noncancerous cell line NIH3T3 (Figure 6a). However, among the polysaccharides tested, IPS2 had higher toxicity (13.32 ± 0.44%) than the other polysaccharides at a high concentration of 1 mg/mL, indicating the biocompatibility of polysaccharides (EPS1, EPS2, and IPS1–4). In terms of cytotoxicity in the cancerous cell line (MDA-MB293), EPS1 exhibited higher cytotoxicity than the other polysaccharides tested. EPS1 showed an IC_50_ concentration of 0.437 mg/mL, while the other polysaccharides did not show an IC_50_ value (Figure 6b). Therefore, EPS1 was selected for further examination via cytotoxicity assay, including staining assay and flow cytometry assay. Similarly, polysaccharides derived from *Trichoderma* species did not affect the growth of a normal cell line (human hepatocytes-LO2), but did inhibit the proliferation of CT26 cells and human breast cancer cell line MCF7 [25,28].

##### Cellular Staining Assay

**Figure 7:**
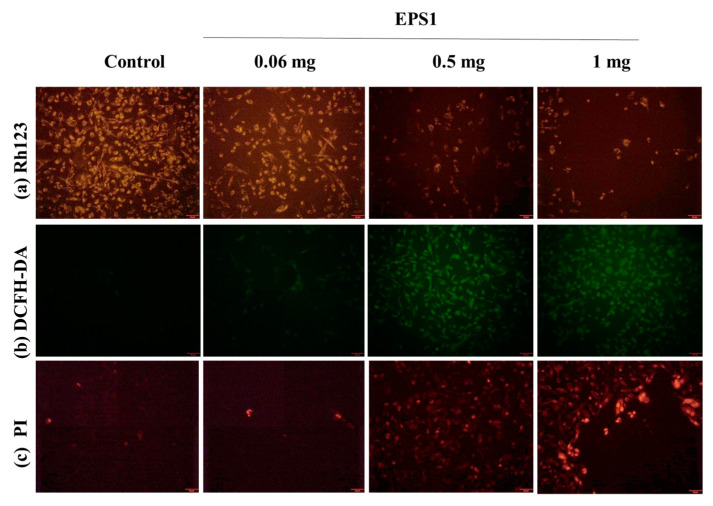
Effect of polysaccharides (EPS1) isolated from *Trichoderma* spp. on cytotoxicity in the MDA-MB231 cell line, demonstrated by fluorescent microscopic staining assay: observation of mitochondrial membrane loss (**a**), reactive oxygen production (**b**), and nucleus damage in membrane-compromised cells (**c**).

Toxicant-induced mitochondrial membrane loss can be studied by using the mitochondrial stain Rh123 [52]. The lower emission of Rh123 in the cells indicates the loss of the mitochondrial membrane potential. Therefore, in the present study, Rh123 was used to observe different concentrations of EPS1-induced mitochondrial membrane loss in MDA-MB293. The results revealed that EPS1 exhibits dose-dependent mitochondrial membrane loss, and this was observed to be higher in the cells treated with 1 mg/mL of EPS1 (Figure 7a). DCFH-DA is a special staining assay used to detect reactive-oxygen-species-mediated oxidative stress in the cells. Higher ROS elevation was found in the cells treated with 0.5 mg/mL and 1 mg/mL of EPS1 (Figure 7b). The PI stain is known to bind to the damaged nucleus in the membrane-compromised cells [53]; hence, PI staining was used to detect the EPS1-induced nucleus damage, and greater nuclear damage was found in the cells treated with 0.5 mg/mL and 1 mg/mL of EPS1 (Figure 7c).

##### AO/EB and Flow Cytometry

**Figure 8:**
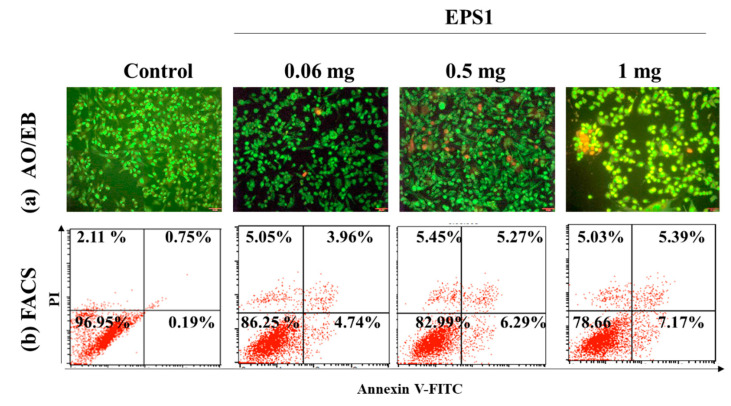
Effect of polysaccharides (EPS1) isolated from *Trichoderma* spp. on cytotoxicity in the MDA-MB231 cell line, demonstrated by AO/EB staining assay (**a**) and Annexin V-FITC- and PI staining-based FACS assay (**b**).

The present study adapted AO/EB staining to observe EPS1-induced apoptosis in the cancerous cell line (MDA-MB293). It is known that the dual staining of AO/EB indicates the stages of cell death, showing live cells in mild green, early apoptotic cells in fluorescent green/orange with chromatin destruction and crescent-like structure of cells, apoptotic cells appear in orange, and dead/necrotic cells emit strong red fluorescence [51]. In the present study, the untreated cells appeared in mild green, while the fluorescent green, orange, and red cells appeared with increasing EPS concentrations. Overall, the results indicate that EPS1 (1 mg/mL) caused high cytotoxicity in the MDA-MB293 cells, which was evident from the greater number of the cells that appeared in strong fluorescent green, orange, and red compared to other treatments and untreated control cells (Figure 8a). Moreover, the FACS results are in agreement with the AO/EB results. The EPS1 (1 mg/mL) treatment exhibited live cells (78.66%), early apoptosis (7.17%), apoptosis (5.39%), and necrosis (5.03%). Overall, the results revealed that the dead cells were found in higher quantities in the cells treated with EPS1 (1 mg/mL) than in other treatments (Figure 8b).

## 4. Conclusions

This work demonstrated the extraction, isolation, purification, antioxidant, enzyme inhibition, and anticancer activities of polysaccharides from *T. harzianum*. A total of six polysaccharides including extracellular polysaccharides (EPS1 and EPS2) and intracellular polysaccharides (IPS1–IPS4) were isolated and characterized by FTIR, SEM, HPLC, and NMR analysis. The FTIR analysis revealed that all of the polysaccharides exhibited the characteristic peaks (hydrogen bonds, C–C group, sugar linkage types) corresponding to polysaccharides. The HPLC results indicated that the polysaccharides (EPS1, EPS2, IPS1, IPS2, and IPS4) contained four sugars including glucose, mannose arabinose, and ribose. EPS1 exhibited (1→4)-linked α-d-glucopyranose, as determined by NMR analysis. Moreover, SEM analysis illustrated the surface morphology of polysaccharides, which indicated that EPS1 and EPS2 had a smooth surface, while the remaining polysaccharides had a microporous structure. All of the polysaccharides showed moderate enzyme inhibition, free radical scavenging, and cytotoxicity, but among them, EPS1 was found to be biocompatible with NIH3T3 cells, with more promising antioxidant, cytotoxic, and enzyme inhibition activity than the other polysaccharides tested in this study. These results revealed that the fungus *T. harzianum* could be a potential source of polysaccharides with unique structures and bioactivity, and further investigation of the molecular elucidation of bioactive properties, along with detailed characterization of other biomolecules in the EPSs and IPSs, is worthwhile. 

## Figures and Tables

**Table 1:** Analysis of the biochemical composition of polysaccharides (EPSs and IPSs) isolated from the *Trichoderma* sp., by spectrophotometric and HPLC assay.

Sample Name	EPS1	EPS2	IPS1	IPS2	IPS3	IPS4
Weight (g)	31.40 ± 1.21	81.60 ± 0.89	27.60 ± 1.84	14.60 ± 0.78	8.10 ± 0.54	2.30 ± 0.12
Yield (%)	63.08 ± 0.15	16.32 ± 0.84	69.49 ± 1.20	10.45 ± 0.78	2.03 ± 0.15	0.57 ± 0.02
Protein (%)	0.31 ± 0.05	0.72 ± 0.07	0.57 ± 0.02	0.79 ± 0.01	0.81 ± 0.07	0.18 ± 0.05
Monosaccharides composition (%)						
Mannose	22.67	20.97	26.61	20.04	35.69	43.98
Ribose	5.78	5.12	6.70	4.98	0	0
Glucose	31.13	40.62	36.42	39.88	39.25	21.22
Galactose	24.22	20.01	11.51	21.99	25.04	34.79
Xylose	0	0	0	0	0	0
Arabinose	16.17	13.25	18.74	13.09	0	0

**Table 2:** ^1^H and ^13^C NMR chemical shifts of polysaccharide (EPS1).

	C1/H1	C2/H2	C3/H3	C4/H4	C5/H5	C6/H6
→4)-α-d-Glc-(1→	99.54/5.23	71.56/3.46	71.05/3.67	76.76/3.59	73.11/3.79	60.02/3.68

## Data Availability

Data is contained within the article or Supplementary Materials.

## Supplementary Materials

The following are available online at www.mdpi.com/article/10.3390/antiox10091372/s1: Figure S1: Determination of monosaccharides by HPLC using the UV detector. Standard chromatogram of standard monosaccharide mix contains mannose (1), ribose (2), glucose (3), galactose (4), xylose (5), and arabinose (6) (a), determination of monosaccharides in EPS2 (b), IPS1 (c), IPS2 (d), IPS3 (e), and IPS4 (d); Figure S2: Structural characterization of EPS2 by NMR spectral analysis: 13C NMR spectrum (a), ^1^H NMR spectrum (b); Figure S3: Structural characterization of IPS (1–4) by NMR spectral analysis: ^1^H NMR spectrum of IPS1 (a), IPS2 (b), IPS3 (c), and IPS4 (d).
