# Supplementary material for: Isolation of Polysaccharides from Trichoderma harzianum with Antioxidant, Anticancer, and Enzyme Inhibition Properties
Source: Antioxidants (Basel). 2021 Aug 28;10(9):1372. doi: 10.3390/antiox10091372 (PMC8471597; doi:10.3390/antiox10091372)
Supplement: Supplementary file 1 [file antioxidants-10-01372-s001.zip › SI.pdf]

Materials

Trichoderma harzianum was previously isolated from the wetland soil by our group [1]. Trifluoroacetic acid (TFA) (Daejung Chemicals & Metals, Korea), DEAE Sepharose Fast Flow (GE Healthcare Life Science, Sweden), 1-phenyl-3-methyl-5-pyrazolone (PMP) (Tokyo Chemical Industry, Japan), Monosaccharides standard kit, 2,2′ -Azino-bis (3-ethylbenzothiazoline-6-sulfonic acid) diammonium salt (ABTS) and 1,2-diphenyl-1-picrylhydrazyl (DPPH) were obtained from Sigma-Aldrich, USA. The noncancerous mice fibroblast (NIH3T3) cell line and cancerous human breast cancer (MDA-MB231) cell line were procured from the Korean Cell Line Bank (KCLB, Seoul, Republic of Korea) and all the other mammalian cell line stains, washing buffer solution and culture medium were obtained from Thermo Fishers Scientific Seoul, Republic of Korea.

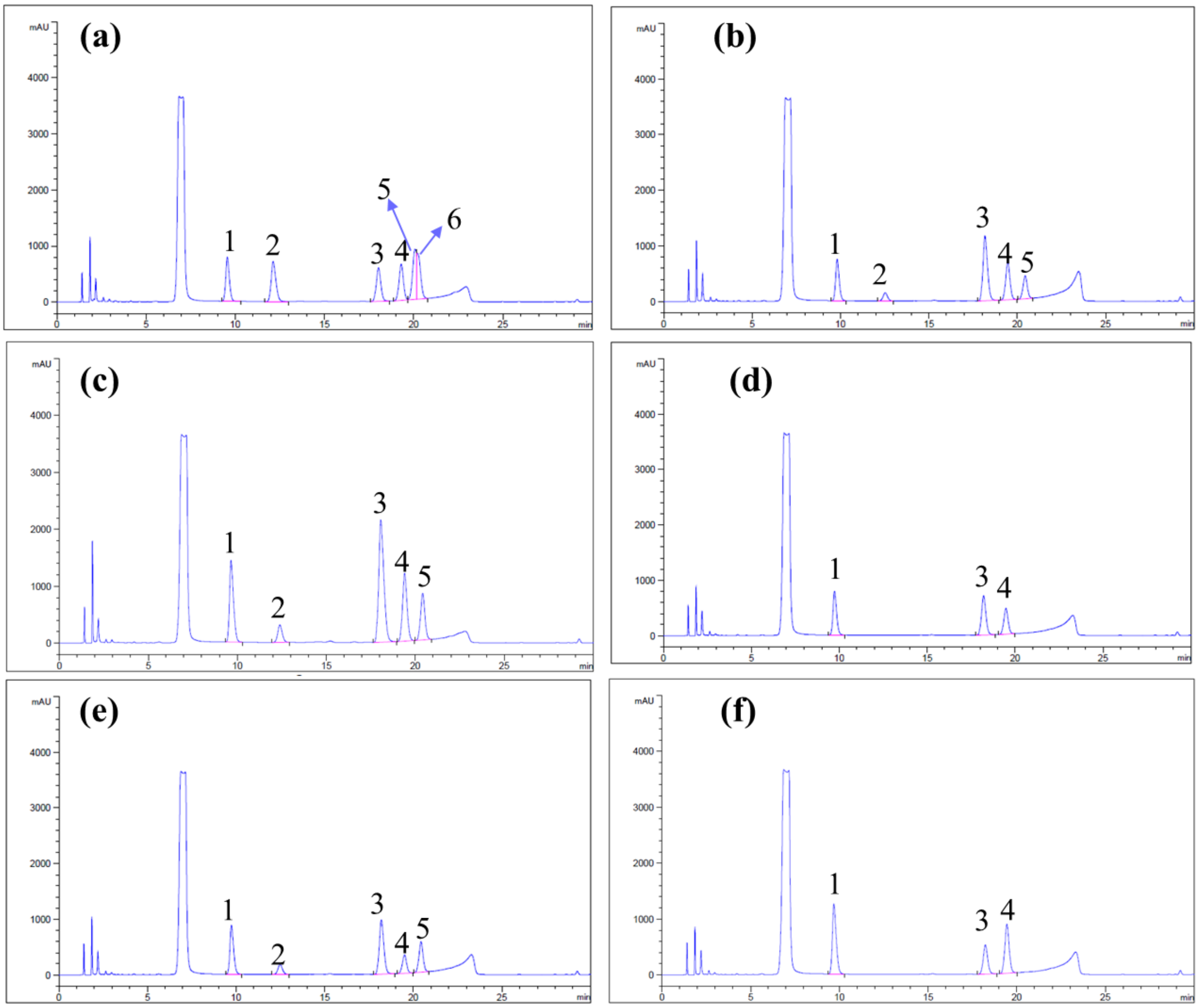

SI.Fig.1. Determination of monosaccharides by HPLC using the UV detector. Standard chromatogram of standard monosaccharide mix contains the mannose-1, ribose-2, glucose-3, galactose-4, xlylose-5 and arabinose-6 (a), determination of monosaccharides in EPS2 (b), IPS1 (c), IPS2 (d), IPS3 (e) and IPS 4 (d).

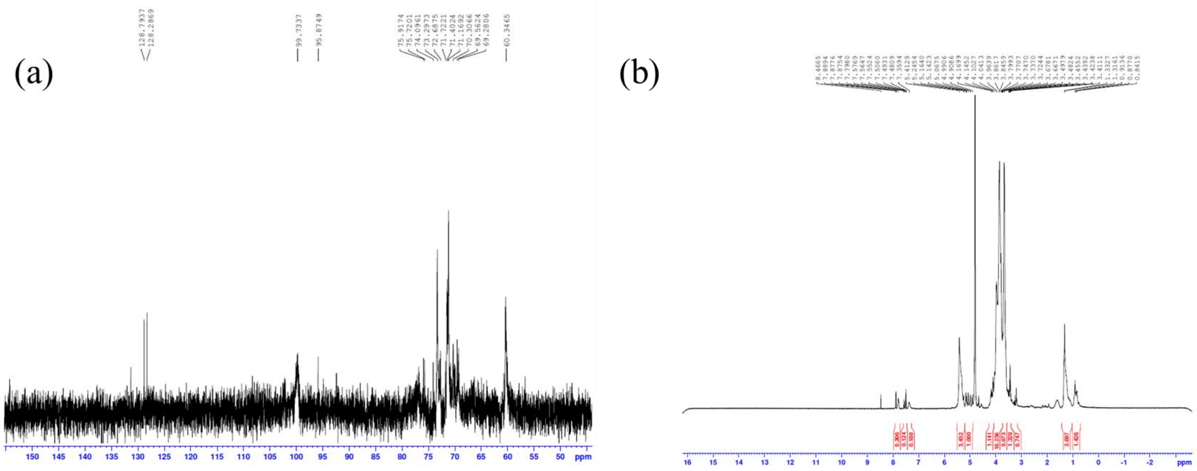

SI.Fig.2. Structural characterization of EPS2 by NMR spectral analysis. ^{13}C NMR spectrum (a), ^{1}H NMR spectrum (b).

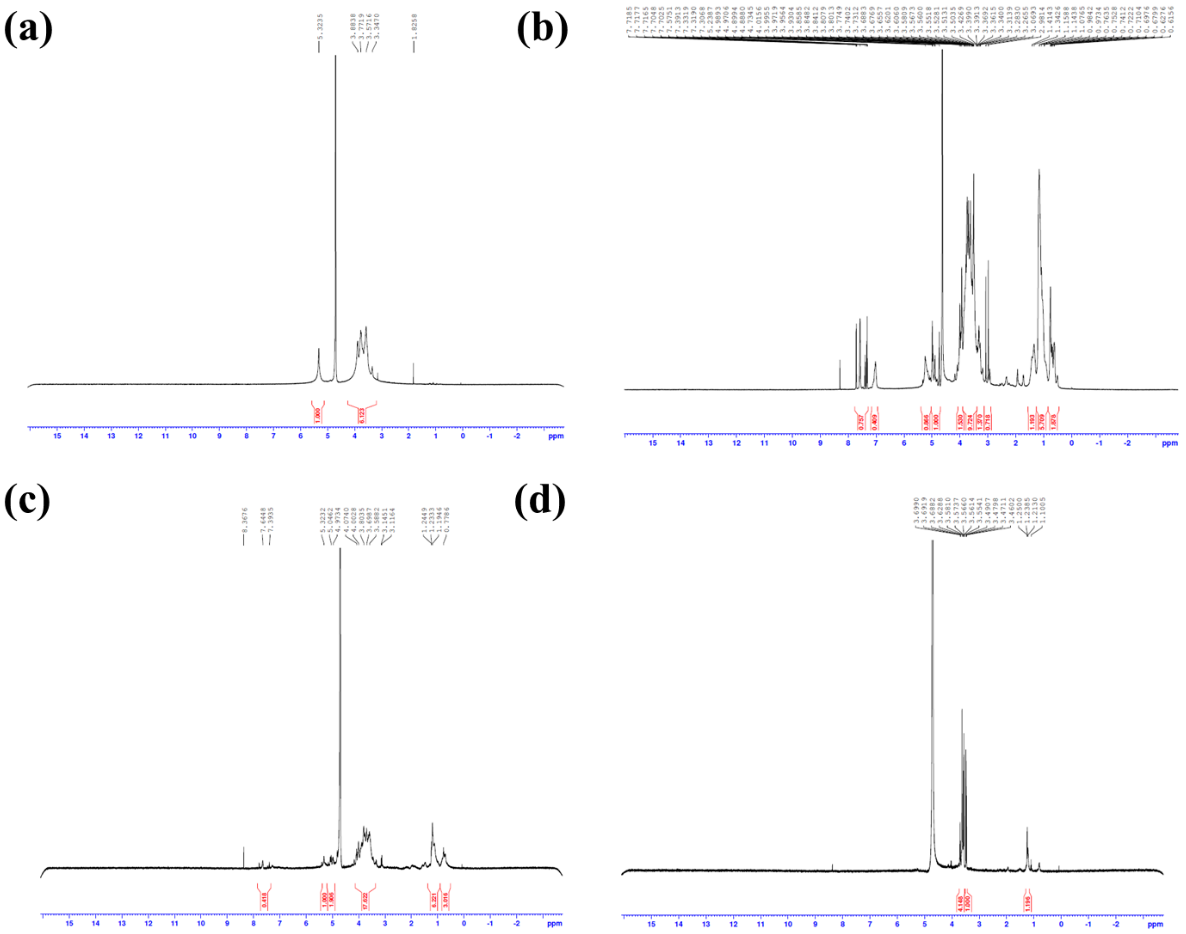

SI.Fig.3. Structural characterization of IPS (1-4) by NMR spectral analysis. ^{1}H NMR spectrum of IPS1 (a), IPS2(b), IPS3 (c) and IPS4 (d).

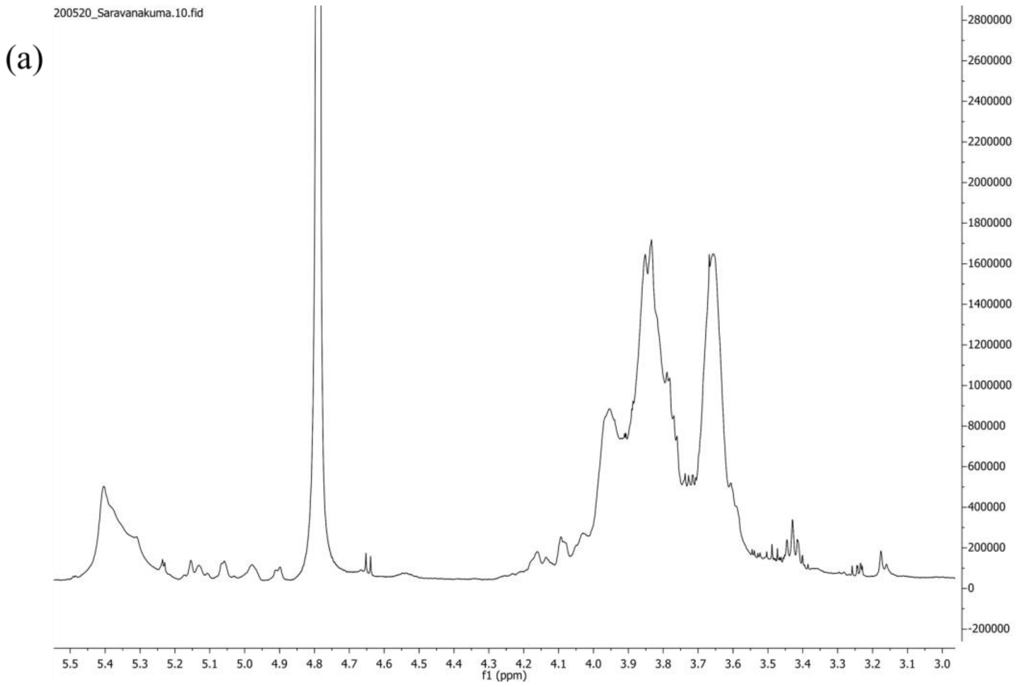

(b)

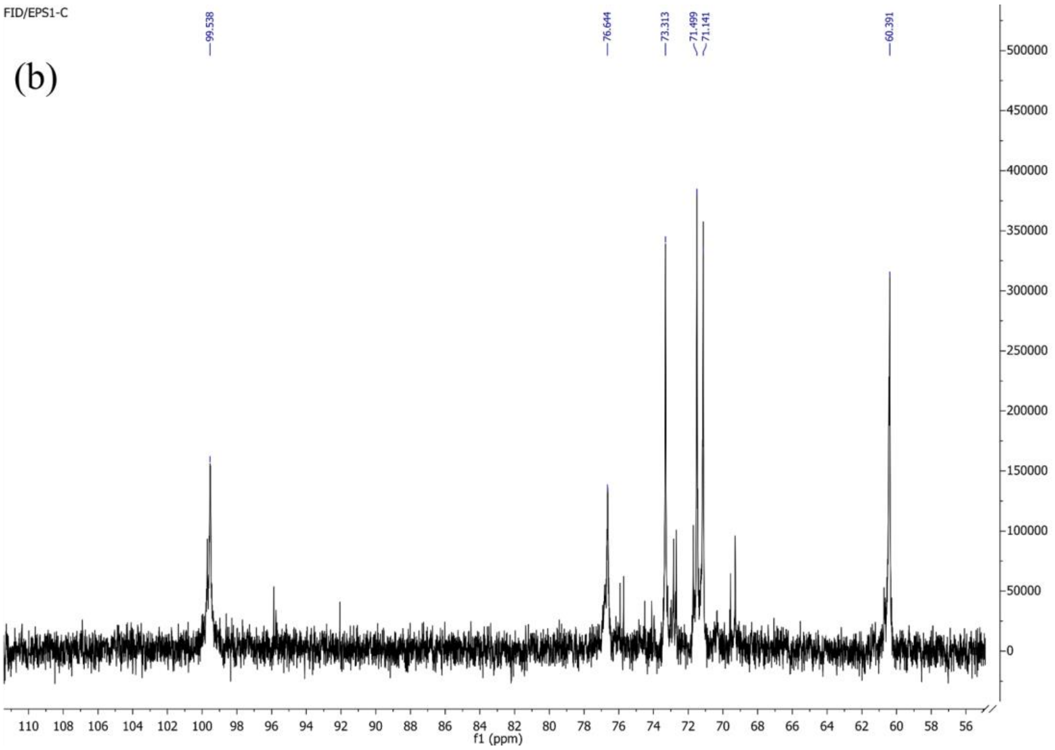
FID/EPS1-C
99.538
76.644
73.313
71.499
71.141
60.391
f1 (ppm)

(c)

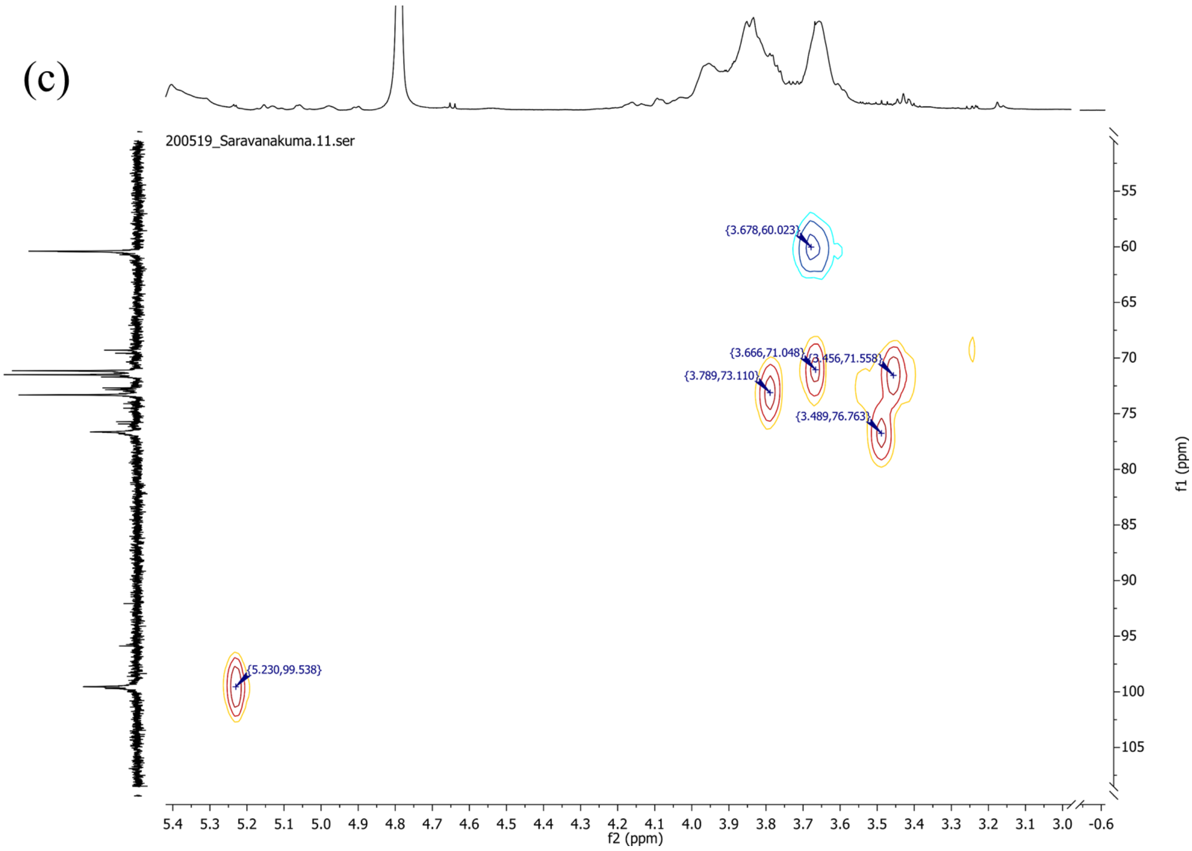
200519_Saravanakuma.11.ser
{3.678,60.023}
{3.666,71.048}
{3.456,71.558}
{3.789,73.110}
{3.489,76.763}
{5.230,99.538}
f2 (ppm)
f1 (ppm)

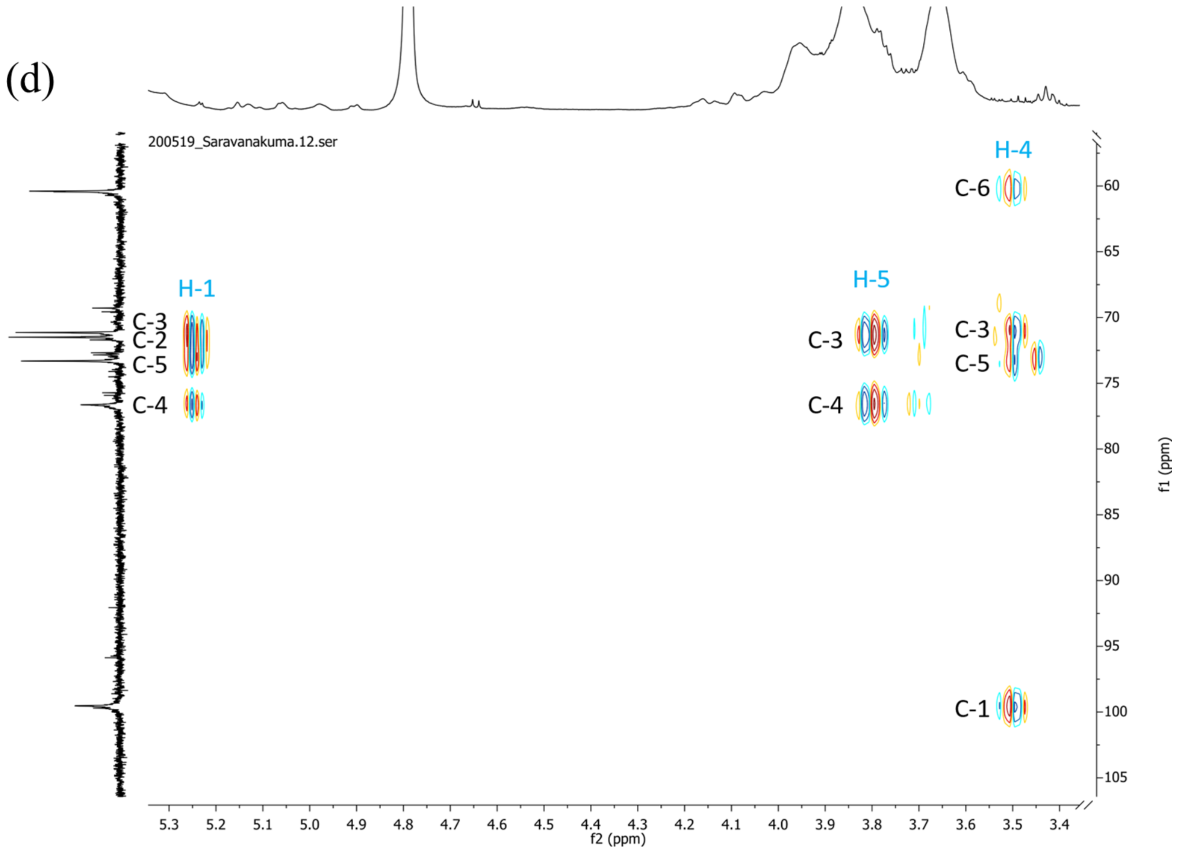

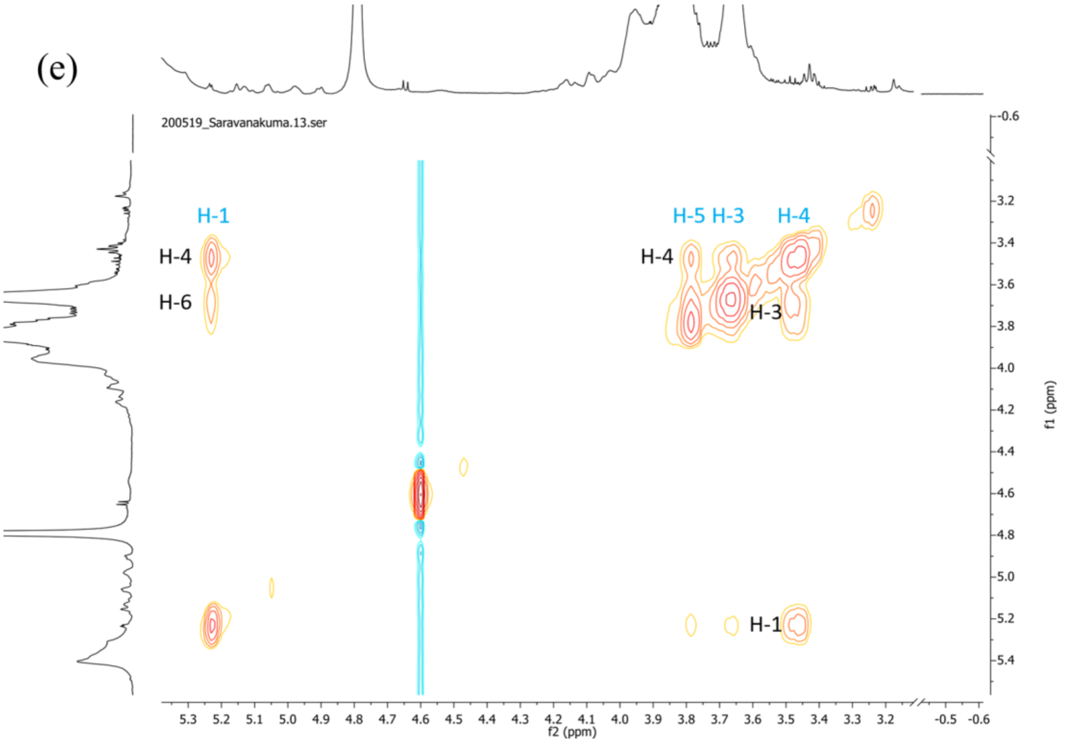

SI.Fig.4. Structural characterization of EPS1 by NMR spectral analysis. 1H NMR spectrum (a), 13C NMR spectrum (b), HSQC spectrum (c), HMBC spectrum (d), and NOESY NMR spectrum (e).

Reference

[1] K. Saravanakumar, M.-H. Wang, Isolation and molecular identification of Trichoderma species from wetland soil and their antagonistic activity against phytopathogens, Physiological and Molecular Plant Pathology 109 (2020) 101458.
